# Differential gene expression and microRNA profile in *corpora allata-corpora cardiaca* of *Aedes aegypti* mosquitoes with weak juvenile hormone signalling

**DOI:** 10.1186/s12864-024-10007-9

**Published:** 2024-01-25

**Authors:** Zhi Qi, Kayvan Etebari, Marcela Nouzova, Fernando G. Noriega, Sassan Asgari

**Affiliations:** 1 Australian Infectious Disease Research Centre, School of Biological Sciences, The University of Queensland, Brisbane, QLD Australia; 2 Institute of Parasitology, Biology Centre CAS, České Budějovice, Czech Republic; 3 Department of Biological Sciences and Biomolecular Sciences Institute, Florida International University, Miami, FL USA; 4 Department of Parasitology, University of South Bohemia, České Budějovice, Czech Republic

**Keywords:** *Aedes aegypti*, Mosquito, Transcriptome, RNA-Seq, Epoxidase, Juvenile hormone, microRNA, *Corpora allata*

## Abstract

The *corpora allata*-*corpora cardiaca* (CA-CC) is an endocrine gland complex that regulates mosquito development and reproduction through the synthesis of juvenile hormone (JH). Epoxidase (Epox) is a key enzyme in the production of JH. We recently utilized CRISPR/Cas9 to establish an *epoxidase-*deficient (*epox*^−/−^) *Aedes aegypti* line. The CA from *epox*^−/−^ mutants do not synthesize epoxidated JH III but methyl farneosate (MF), a weak agonist of the JH receptor, and therefore have reduced JH signalling. Illumina sequencing was used to examine the differences in gene expression between the CA-CC from wild type (*WT*) and *epox*^*−/−*^ adult female mosquitoes. From 18,034 identified genes, 317 were significantly differentially expressed. These genes are involved in many biological processes, including the regulation of cell proliferation and apoptosis, energy metabolism, and nutritional uptake. In addition, the same CA-CC samples were also used to examine the microRNA (miRNA) profiles of *epox*^*−/−*^ and *WT* mosquitoes. A total of 197 miRNAs were detected, 24 of which were differentially regulated in *epox*^−/−^ mutants. miRNA binding sites for these particular miRNAs were identified using an *in silico* approach; they target a total of 101 differentially expressed genes. Our results suggest that a lack of epoxidase, besides affecting JH synthesis, results in the diminishing of JH signalling that have significant effects on *Ae. aegypti* CA-CC transcriptome profiles, as well as its miRNA repertoire.

**Supplementary Information:**

The online version contains supplementary material available at 10.1186/s12864-024-10007-9.

## Introduction

The *corpora allata* (CA) is the site of synthesis of juvenile hormone (JH), an essential sesquiterpenoid that controls development and reproduction in insects [1]; while the *corpora cardiaca* (CC) is a neurohemal/endocrine gland that synthesizes neuropeptides such as adipokinetic hormones (AKHs) that regulate metabolism [2]. In *Aedes aegypti* mosquitoes, a deficiency of JH in the adult stage results in ovarian follicular resorption and reduced fecundity [3]. In mosquitoes, the last two steps of JH III biosynthesis involve metabolism of two precursors: farnesoic acid (FA) and methyl farnesoate (MF). Two enzymes are involved in these two steps. JH methyltransferase (JHAMT) synthesizes MF from FA, and methyl farnesoate epoxidase (Epox) further oxidizes MF to JH III [1].

CRISPR/Cas9 was employed to generate an epoxidase-deficient (*epox*^*−/−*^*) Ae. aegypti* line [4]. The CA from *epox*^*−/−*^ mutants do not synthesize epoxidated JH III but MF, a weak agonist of the JH receptor, and therefore these mutants have reduced JH signalling [4]. The mutant mosquitoes complete their life cycle, but while *epox*^*−/−*^ adults were fertile, the reproductive performance of both sexes was dramatically reduced [4].

MicroRNAs (miRNAs) are a class of ~ 22 nt small RNAs that regulate gene expression at both the translational and transcriptional levels. They guide the RNA-induced silencing complex (RISC) to their mRNA targets, modulating gene expression at the post-transcriptional level [5]. Further, miRNAs can potentially regulate heterochromatin formation and trigger knock down of gene expression at longer timescales [6]. In addition, miRNAs may up-regulate target gene translation through different pathways, such as inhibiting the binding of RNA-degrading protein, assisting poly-A tail loop formation, and recruiting activation factors [7–9].

Specific miRNAs in the CA-CC might contribute to the modulation of JH synthesis. Our previous analysis of CA-CC miRNA profiles in *Ae. aegypti* showed that many miRNAs were differentially expressed among diverse developmental stages of the mosquito, with different levels of JH biosynthesis [10]. To address the question of how a reduction of JH signalling might influence signalling to, from and within the CA-CC complex, we generated transcriptome libraries for both WT and *epox*^*−/−*^*Ae. aegypti* CA-CC, and investigated the differential expression of genes and miRNAs. These results might help to identify CA-CC gene networks that might participate in regulating development and reproductive processes.

## Materials and methods

### Insect collection and RNA extraction

*Aedes aegypti* mosquitoes (Orlando) were raised at 28 °C, 80% relative humidity, and 16 h light/8 hours dark photoperiod. Larvae were provided Tetramin tropical fish food (cat #16,152, Tetra). Adult mosquitoes were offered 10% sugar water *ad libitum*. Four-to-five-day old female mosquitoes were artificially fed pig blood equilibrated to 37 °C. ATP was added to the blood meal to a final concentration of 1 mM immediately before use.

An *epox*^*−/−*^ mutant line generated previously by CRISPR/Cas9 via embryonic microinjection [4] was used in this study. *Corpora allata* were dissected from *epox*^*−/−*^ and *WT* 3-4-day-old sugar-fed adult female mosquitoes in three replicates. Each replicate comprised of 50 CA-CCs. Total RNA was extracted and DNase-treated using a Norgen Biotek’s total RNA purification kit. Total RNA was treated with DNase I according to Norgen Biotek’s instructions. RNA samples were sequenced by LC Sciences (Texas, USA).

### RNA-Seq data analysis

The CLC Genomic Workbench v20.0.2 (QIAGEN) was used for removing adapter sequences/low-quality reads and processing the sequencing data. Transcriptome and small RNA data were generated from Illumina sequencing. Small RNA data were trimmed using the following adapters: Illumina Truseq Small RNA 3’ Adapter (RA3) (TGGAATTCTCGGGTGCCAAGG), and mRNA data were trimmed using the Automatic read-through adaptor trimming function in CLC Genomics workbench. Low quality reads were discarded. Trimmed miRNA reads were mapped to the latest *Ae. aegypti* reference genome downloaded from NCBI Reference Sequence Database (GCF_002204525.1). For miRNA sequencing data, we applied a minimum length fraction = 0.5, similarity fraction = 0.8, match score = 1, and mismatch cost = 2 as matching criteria. miRNAs were identified using resources from miRBase (www.miRBase.org) and our previously generated extended *Ae. aegypti* miRNA profile [10]. Trimmed transcriptome reads were also mapped and assembled using the *Ae. aegypti* reference genome downloaded from the NCBI Reference Sequence Database (GCF_002204525.1). For mRNA RNA-Seq analysis, we applied a mismatch cost = 2, insertion and deletion costs = 3, length and similarity fractions = 0.8, maximum number of hits for a read = 10, strand setting = Both, library type setting = Bulk, and minimum read count fusion gene table = 5. Low quality reads (quality score < 0.05) and reads with more than two ambiguous nucleotides were discarded.

Gene Ontology (GO) analysis was performed by uploading all the differentially expressed genes to the Blast2GO bioinformatics platform for functional annotation analysis [11]. We utilised BLAST, InterProScan [12], enzyme classification codes (EC), and EggNOG [13] to determine the GO terms associated with the differentially expressed sequences. More abundant terms were computed for each category of molecular function, biological process, and cellular components.

An enrichment analysis using Fisher’s Exact Test was conducted, using all AaegL5.0 annotated genes as the reference dataset. This analysis was performed with the FatiGO package, which is integrated into Blast2GO. Overrepresented and underrepresented terms were identified if their adjusted *p* value was less than 0.05. A dot plot chart was generated to visualise the 30 most enriched GO terms of both upregulated and downregulated genes. These 3-dimensional charts represent the GO annotation term on the Y-axis, the gene ratio (Nr Test / [Nr Test + Not Annot Test]) on the X-axis, and the number of test sequences in the set as the dot size. Adjusted *p* values are indicated using a colour scheme.

To identify potential miRNA binding sites within all differentially expressed *Ae. aegypti* genes, we employed three distinct algorithms: RNA22 [14], miRanda [15], and RNAhybrid [16]. RNAhybrid is a tool utilised for determining the normalized minimum free energy (MFE) of hybridization between miRNA and their mRNA target genes. The small RNA sequence is paired with the most compatible section of the mRNA. Within the seed region (nucleotides 2–8), we excluded G:U pairings, and enforced the presence of a helix in the miRNA-target duplexes. An allowance of up to five unpaired nucleotides on either side of an internal loop was permitted. While miRanda considers matching throughout the entire miRNA sequence, we ran the program in strict mode, prioritizing strict 5’ seed pairing. The seed region was assigned additional significance by assigning higher value to matches in this region. RNA22 v.2, on the other hand, is a target prediction program that relies on patterns. Initially, it searches for reverse complement sites within a given mRNA sequence and identifies hot spots. Subsequently, the algorithm searches for miRNAs that are likely to bind to these sites. In our analysis, we allowed a maximum of one mismatch in the seed region and a minimum of 12 nucleotide matches in the entire binding site. We set the thresholds for sensitivity and specificity at 63% and 61%, respectively. Two-model analysis based on minimum free energy (MFE), and number of binding sites were used to identify potential targets [17]. To increase the level of confidence, we selected those binding sites that were predicted at least by two out of three of the algorithms.

## Results and discussion

Earlier studies described that miRNAs are involved in the regulation of metamorphosis and related hormone signalling [18]. Previous research on CA-CC transcriptome biology emphasized the relation between CA-CC miRNAs and JH biosynthetic activity; as well as the importance of establishing CA-CC transcriptome libraries [10, 19, 20]. The role of JH in controlling reproductive trade-offs has been extensively studied in female mosquitoes. Depending on nutritional and hormonal homeostasis, the final number of eggs that develop can be adjusted at different times during oogenesis. The JH titre (and therefore JH signalling) is fine-tuned to match oogenesis to the nutritional status [3, 21]. The *epox*^−/−^ mutant line is an excellent model for the study of CA-CC gene expression in mosquitoes; the CA of epoxidase mutants synthesize and release MF instead of JH III. These mutant females, with low JH signalling, produce fewer eggs. Comparing miRNA expression profiles between the two lines enabled us to screen for JH-responsive miRNAs and genes in the mosquito CA-CC that are differentially expressed in the epoxidase mutants allowing to further understand how insects might compensate for low JH titre and signalling.

### miRNA profiling of CA-CC of *epox*^*-/-*^ mutant and *WT* mosquitoes

Illumina small RNA deep sequencing platform was used to produce three biological replicates of small RNA profiles of CA-CC *epox*^*−/−*^ mutant and *WT* mosquitoes. We acquired a total of 34,370,595 small RNA raw reads for *epox*^*−/−*^ mutant libraries and 39,751,144 for WT mosquitoes (Table S1). Less than 0.05% of reads were discarded in the different libraries due to their low-quality score or lack of adapter sequence, stressing the high quality of the sequencing samples. We detected 197 annotated *Ae. aegypti* miRNAs present on miRBase and our previously annotated *Ae. aegypti* miRNAs [10].

**Fig. 1:**
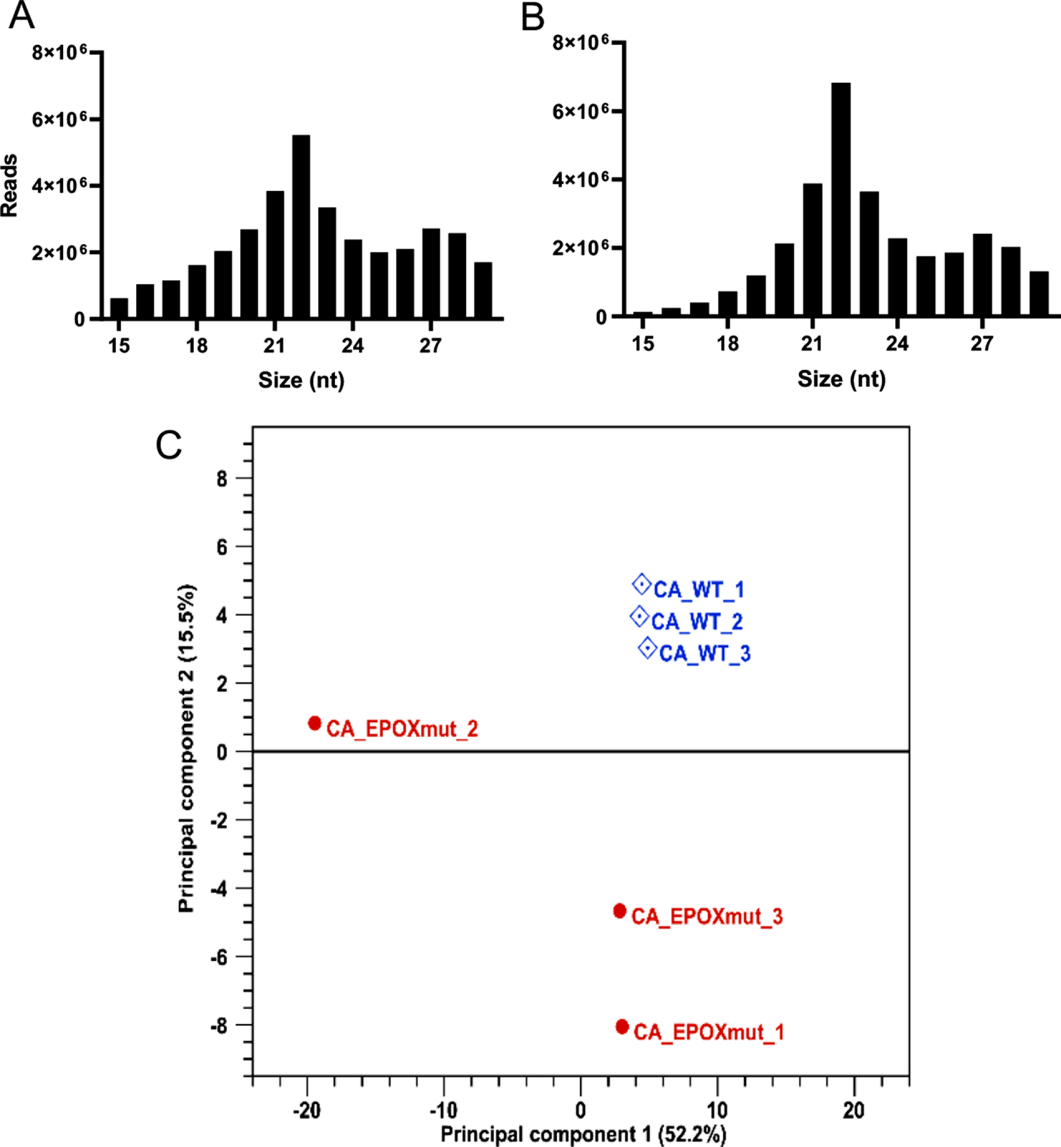
Read distributions and Principal Component Analysis of RNA-Seq data. Distribution of trimmed reads of *WT* (**A**) and *epox*^*−/−*^ samples (**B**). (**C**) Principal Component Analysis of *WT* and *epox*^−/−^ mosquitoes based on small RNA library reads. The plot projects samples onto two-dimensional space with X and Y axes being first and second principal components. The samples are clustered by the similarity of the abundancy of their miRNA

We observed a significant spike in total read numbers at 21–23 nucleotides, which corresponds to the typical length of miRNAs and siRNAs (Fig. 1A and B). Additionally, a smaller peak was observed at 27–29, likely corresponding to PIWI-interacting RNAs (piRNAs), which are commonly present in small RNA libraries of insects (reviewed in [5]).

### Differential expression of miRNAs between CA-CC of *epox*^*-/-*^ mutant and *WT* mosquitoes

Our previous research showed that miRNA repertoires were different when JH biosynthesis varies [10]. Given the JH biosynthetic differences between the *WT* and *epox*^*−/−*^ mosquitoes, we expected differences in the expression of miRNAs in the CA-CC of both lines. To test this hypothesis, we conducted a comparative study of the differentially abundant miRNAs of CA-CC between *WT* and *epox*^*−/−*^ mutant. Principal Component Analysis (PCA) was conducted on miRNA libraries from *WT* and *epox*^−/−^*Ae. aegypti*. All three *WT* samples had similar profiles and are in one cluster. Two *epox*^−/−^ mutants are in one cluster, while one *epox*^−/−^ mutant sample (EPOXmut_2) is located outside the cluster due to its different miRNA profile (Fig. 1C).

**Fig. 2:**
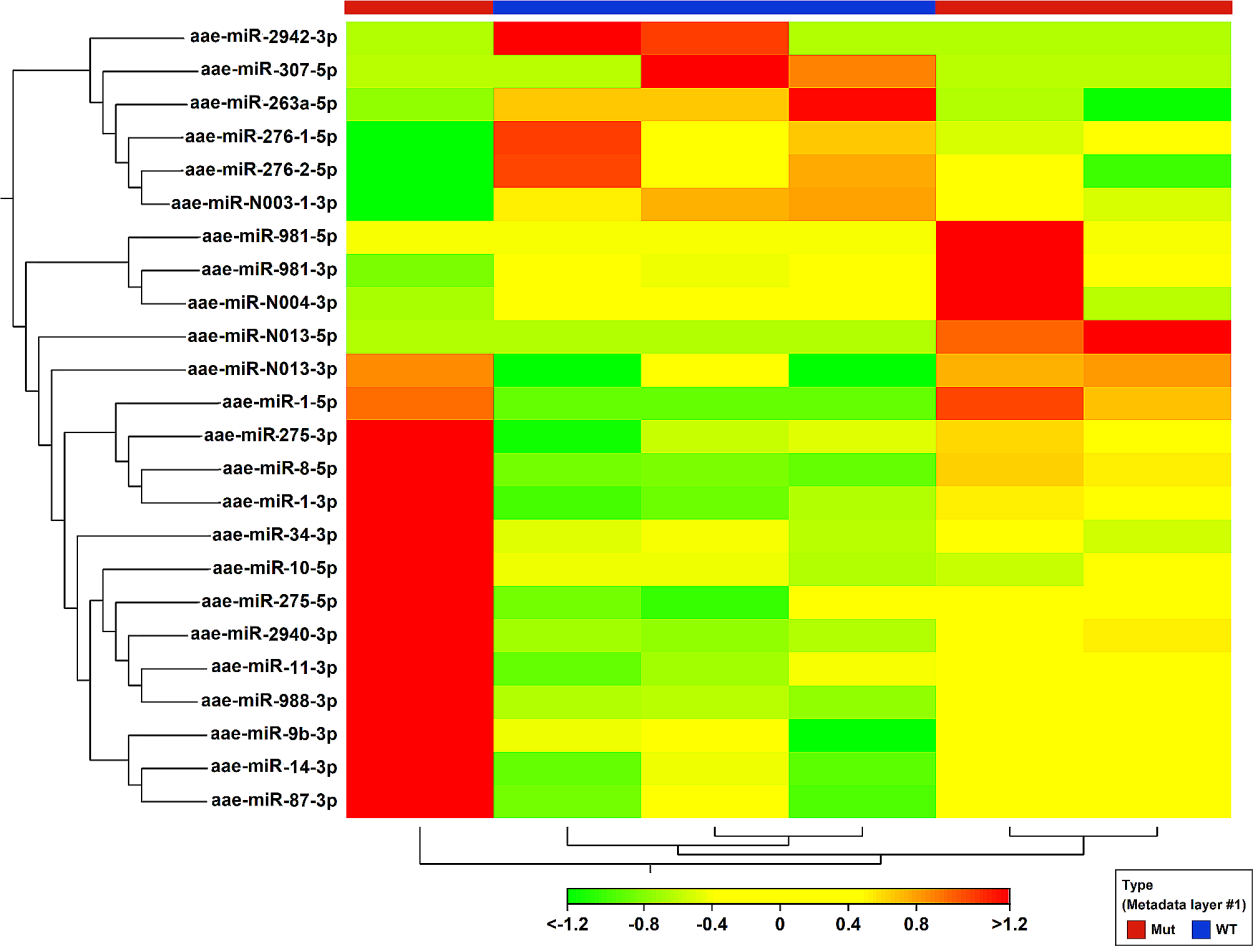
Differentially expressed miRNAs between WT and epox−/− mutant

**Fig. 3:**
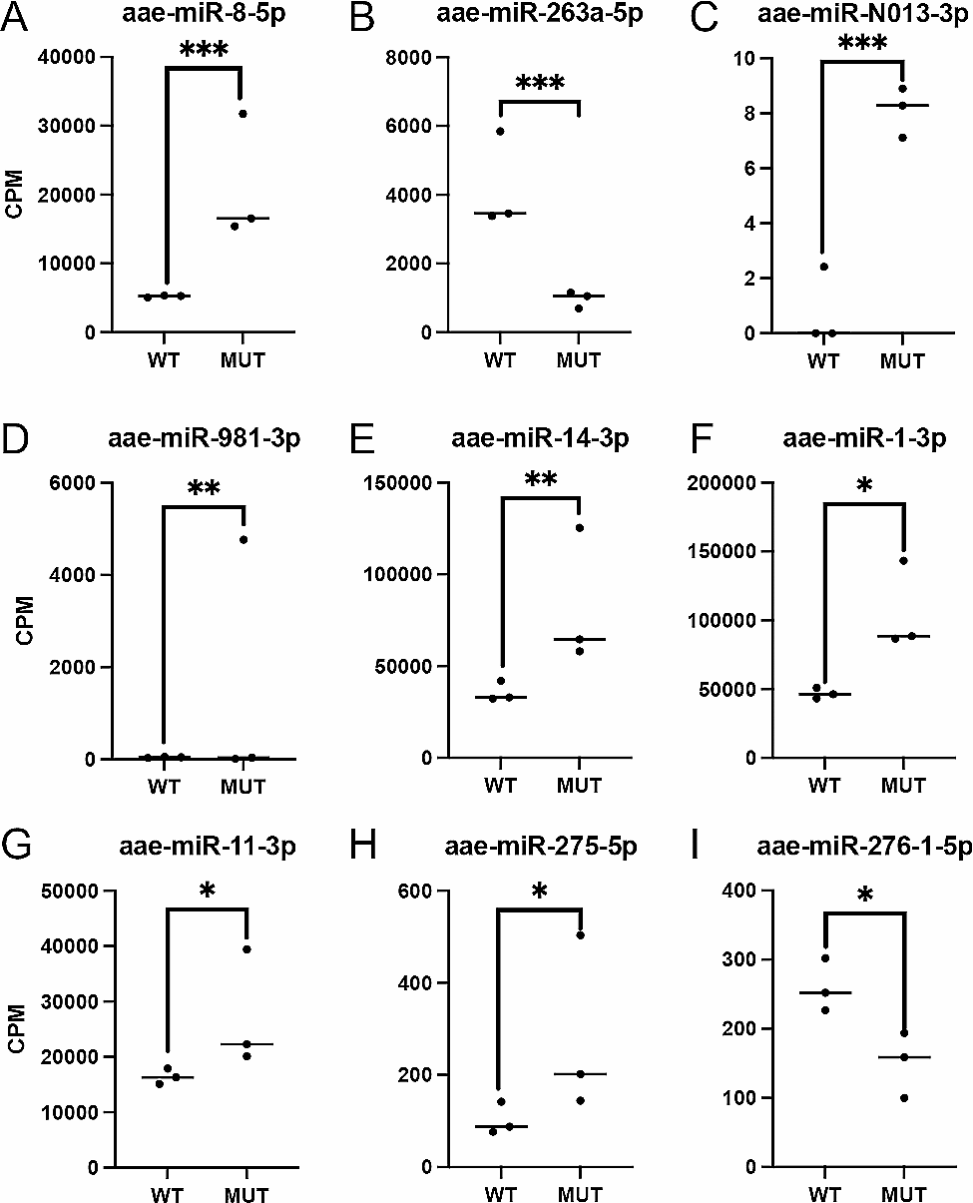
Differentially expressed miRNAs between *WT* and *epox*−/− mutant. (**A-I**) DE miRNAs with the lowest *p* values (*p* < 0.005, **p < 0.0005, ***p < 0.000050) are shown. Each data point represents a biological replicate. CPM, count per million

Our analysis identified 24 differentially abundant miRNAs (Fig. 2). Among these miRNAs, aae-miR-N013-5p, aae-miR-1-5p, and aae-miR-981-5p were the most up-regulated miRNAs in the *epox*^*−/−*^ mosquitoes; with fold changes (FCs) of 169, 116, and 67, respectively. In contrast, the most downregulated miRNAs were miR-307-5p, miR-2942-3p, and miR-1890-5p, with FCs of -94, -48 and − 41, respectively. Data analysis revealed that the knockout of *epox* led to statistically significant changes in the abundance of certain miRNAs, as evidenced by a normalized fold change above 1.5 and *p <* 0.05 (Fig. 3). The most significantly downregulated miRNA was miR-263a-5p (Fig. 3B), and the most significantly up-regulated miRNAs were miR-8-5p and miR-N013-3p, with *p* < 0.00005 (Fig. 3A and C). miRNA profiles were generated for all small RNA libraries, with a minimum threshold of five reads for each miRNA. The count of identified mature miRNA varied between 0 and 143,307 counts per million (CPM) in each sample. Among the significantly differentially expressed miRNAs, the top three with the highest max group means include miR-1-3p, miR-14-3p, and miR-275-3p, with max group means of 284,424, 218,293, 109,722, and *p* value of 1.3E-03, 1.4E-04, 3.7E-02, respectively.

In our previous study [10], 72 differentially expressed miRNAs were found between *Ae. aegypti* pupa (CA inactive, CA-^pup^) and sugar-fed adult (CA active, CA + ^sug^) while 23 miRNAs were found differentially expressed between blood-fed (CA inactive, CA-^bf^) and CA + ^sug^. Similar to *epox*^−/−^, JH synthesis is very low in CA-^pup^ and CA-^bf^. A comparative analysis between the differentially expressed miRNAs between CA-^pup^, CA-^bf^, CA + ^sug^, *epox*^−/−^ and *WT* was conducted (Fig. S1). CA-^pup^, CA-^bf^ and *epox*^−/−^ have low JH biosynthesis, while CA + ^sug^ and *WT* are actively synthesizing JH. Three up-regulated miRNAs (aae-miR-1-3p, aae-miR-2940-3p, aae-miR-34-3p) and one down-regulated miRNA in *epox*^−/−^ (aae-miR-263a-5p) showed similar trend in CA-^pup^. On the other hand, one up-regulated (aae-miR-10-5p) and one down-regulated (aae-miR-276-2-5p) miRNA were found to show similar trend in CA-^bf^ and *epox*^−/−^*Ae. aegypti* (Tables S2, S3).

Interestingly, miR-N013-5p, miR-1-5p, and miR-981-5p were only detected in *epox*^*−/−*^ samples, whereas miR-307-5p and miR-2942-3p were exclusively detected in *WT* samples (Table S3). Knowledge about the functions of these miRNAs in mosquitoes is limited. It has been reported that miR-1 maintains muscle fibre integrity during rapid growth, and its depletion can cause lethality in *Drosophila melanogaster* [22]. Previous studies suggested that miR-1 achieves this by repressing the function of the vacuolar adenosine triphosphatase (V-ATPase) complex [23].

Depletion of miR-307-5p may contribute to a small body size observed in *epox*^*−/−*^*Ae. aegypti* [4]; as previous research has demonstrated that the expression level of miR-307 varies across insect developmental stages, suggesting a potential regulatory effect of JH on this miRNA. Target genes of miR-307 include genes such as *sr, fkh, Stat92E, CG32467, Or42a*, and *kkv*, which are involved in insect cell growth, neuronal and reproductive organ development, and chitin-based cuticle synthesis [24]. *Heat shock protein 70* (*Hsp*70) is a stress-responsive gene that is up-regulated under different stress conditions [25, 26]; previous research suggested that *Hsp70* may contain binding sites for miR-307-5p [26], however, a direct relationship between miR-307 and CA activity has not been previously reported.

miR-2942-3p, which is absent in *epox*^*−/−*^ mutants, is highly expressed in mosquito larvae, and gradually decreases in pupae and adults. miR-2942-3p abundance is positively correlated to *Aedes albopictus* eclosion success [27]. It has been reported that miR-2942-3p facilitates *Ae. albopictus* hatching and pupation, and it is downregulated in diapausing larvae [27, 28]. Although diapause has not been described in *Ae. aegypti*, low JH expression is one of the inductive factors of adult insect reproductive diapause [29, 30] and embryonic diapause in *Ae. albopictus* [31].

Several miRNAs that are differentially expressed in *epox*^*−/−*^ CA-CC have been suggested to play a role in regulating the ecdysone pathway. For instance, miR-14 represses the expression of the ecdysone receptor gene (*EcR*) [32]. Its overexpression in *epox*^*−/−*^ suggests that JH may be involved in regulating ecdysone signalling in the CA-CC. Let-7-5p is up-regulated in *epox*^*−/−*^ but is not statistically significant (not included in Table S3). This well-studied miRNA is also suppressed by JH. Let-7, in turn, suppresses *Kr-h1*, a transcription factor central in JH signalling [33].

Our data showed miR-87-3p is among the modulated miRNAs (Table S3). It was significantly up-regulated in *epox*^*−/−*^ CA-CC by 1.8-fold. This miRNA targets the *Tramtrack69* gene that suppresses *D. melanogaster* progenitor cell differentiation [34]. Our studies also showed that miR-11-3p is significantly overexpressed in *epox*^*−/−*^ mosquitoes by 1.65-fold, with *p* < 0.0016 (Table S3). In previous studies, miR-11 depleted mutant *D. melanogaster* displayed defects in the central nervous system; and double mutations with miR-6, resulted in lethality [35]. However, the role of this miRNA in CA-CC biology remains to be elucidated.

Several miRNAs playing crucial roles in regulating insect reproduction were also found to be differentially expressed in our study. For instance, miR-275, which is up-regulated in *epox*^*−/−*^ mutant mosquitoes by 1.63-fold (miR-275-3p) and 2.77-fold (miR-275-5p) (Table S3), targets sarco/endoplasmic reticulum Ca^2+^ adenosine triphosphatase (*SERCA*), which consequently regulates Notch cell signalling in *Ae. aegypti* [36]. The SERCA pump is key regulator of cellular calcium homeostasis, a major factor in the regulation of JH biosynthesis in the CA of insects [37]. In addition, miR-8, which was up-regulated by 4-folds in *epox*^*−/−*^ (Table S3), is highly expressed in the CA of *D. melanogaster* and exerts a positive effect on cell growth and JH biosynthesis [20]. Inhibition of miR-8 in *D. melanogaster* decreased *Jhamt* expression; on the contrary, overexpression of miR-8 increased *Jhamt* expression [20]. In *D. melanogaster*, loss of miR-8 resulted in a significant decrease in CA cell nucleus size and expression of Jhamt, suggesting that miR-8 is required for CA cell growth and JH biosynthesis. miR-8 appears to perform diverse functions in growth control of different cell types; it increases body size in *D. melanogaster*, and it is inhibited by 20-hydroxyecdysone (20E) [38]. Moreover, miR-8 represses Secreted Wg-interacting molecule (Swim) and positively regulates *Ae. aegypti* reproduction by increasing its fat body mass [39]. In humans, miR-8 positively regulates cell growth by activating PI3K and thus promoting insulin/IGF-1 signalling (IIS) [40]. The cockroach CA experiences significant cell size changes in mated females associated with concomitant changes in JH production [41, 42]; miR-8 acts as a positive regulator of CA cell size, although there is no evidence that increase of CA size in mosquitoes might be a major factor for JH biosynthesis [41].

Furthermore, miR-34-3p, which was up-regulated in *epox*^*−/−*^ mutant mosquitoes (Table S3), targets the insulin receptors *InR1* and *InR2* in *Nilaparvata lugens* [43, 44]. Co-repression of *InR1* and *InR2* by miR-34 significantly increased the number of eggs deposited by *N. lugens* [44]. The stimulatory role of insulin in JH synthesis has been well described in *D. melanogaster* [20, 45] and mosquitoes [46, 47].

### Differential expression of mRNAs between CA-CC of *WT* and *epox*^*-/-*^ mosquitoes

**Fig. 4:**
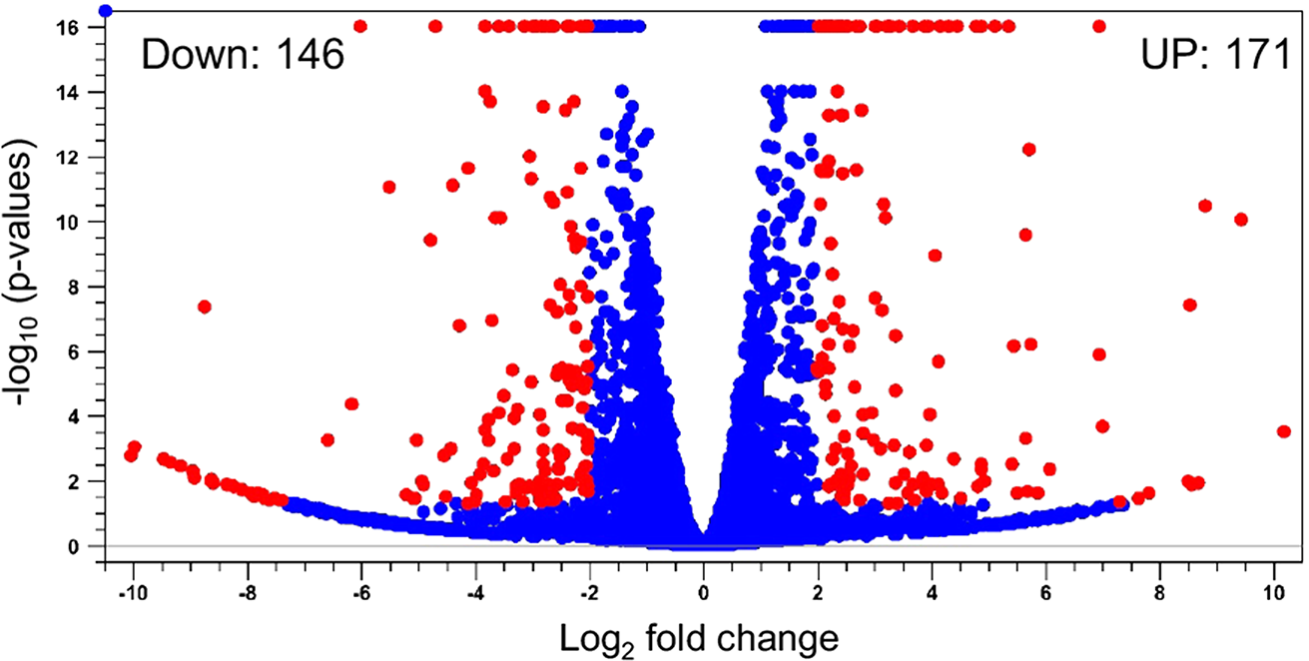
Volcano plot of CA-CC genes of *epox*^*−/−*^ mutant and *WT Ae. aegypti* mosquitoes. Each dot is a recognized sequence. Red: sequences matching significance criteria (Fold change > 2, FDR *p* ≤ 0.05) for differential gene expression

To investigate which CA-CC genes are affected by decreased JH signaling, we conducted a comparative analysis of the transcriptome of CA-CC of *epox*^*−/−*^ and *WT* mosquitoes. The total number of trimmed reads in each sample varied from 130,018,262 to 153,470,316 (Table S4). Reads mapped in pairs were between 84.33 and 91.25%, indicating high quality of sequencing. The transcriptome analysis revealed that out of the 18,034 total identified *Ae. aegypti* genes, 317 were differentially expressed (Table S5). There were 171 up-regulated and 146 down-regulated genes in *epox*^*−/−*^ mutants (Fig. 4).

**Fig. 5:**
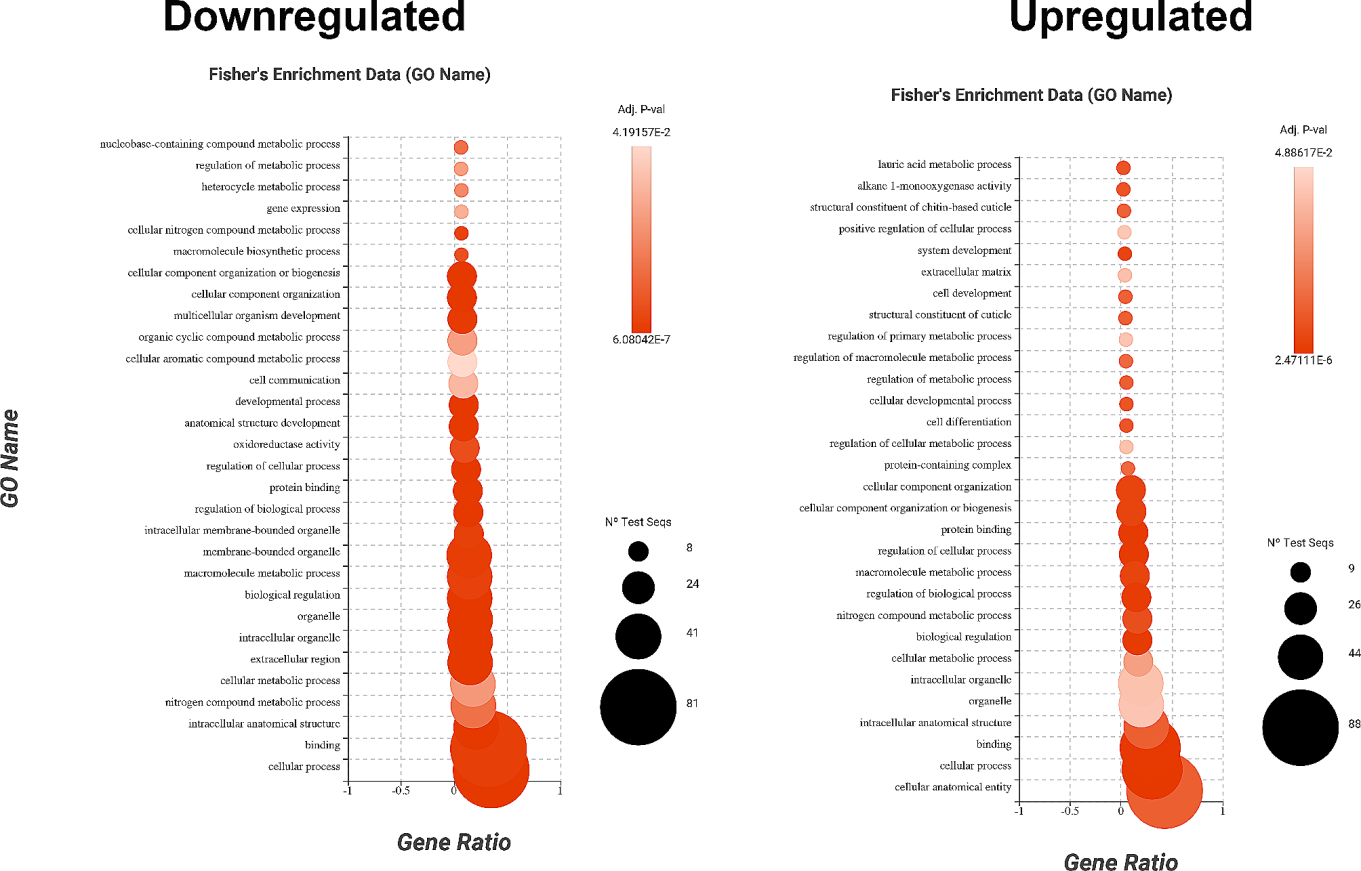
Gene Ontology (GO) analysis representing the 30 most abundant GO terms of differentially expressed genes in *epox*−/− mutants. The dot plot is a combination of biological processes, molecular functions, and cellular components

A dot plot was produced showing the Gene Ontology (GO) terms for biological process, molecular function, and cellular component of the DEGs. In total, 60 and 77 GO terms were enriched in up- and down-regulated genes, respectively. Among these GO terms, “extracellular region” (GO:0005576) was the most significant over-represented GO term (Adj. *p* = 6.08E-07) in the set of genes which were down-regulated in *epox*^*−/−*^ mutant samples. GO terms of “cellular process” (GO:0009987) were allocated to 63 genes and were significantly underrepresented in upregulated genes (Adj. *p =* 2.47E-06). Some of the most abundant GO terms which were significantly enriched in this study are cellular process, cellular anatomical entity, and binding (Fig. 5). Genes with annotated GO terms of nervous system development (GO:0007399), neuron differentiation (GO:0030182) and neurogenesis (GO:0022008) were among the differentially expressed genes (Tables S6, S7).

Among DEGs, *Trypsin epsilon (EpsilonTry*, FDR *p* = 3.59E-11), *nose resistant to fluoxetine protein 6* (*Nrf-6*, FDR *p* = 0.01) and *octopamine receptor 1* (*Octr-1*, FDR *p* = 0.01) were the most upregulated, with Log_2_ FCs increased by 444, 375, and 375, respectively (Table S5). It is noteworthy to mention that *Nrf-6* and *Octr-1* were barely detectable (RPKM = 0) in *WT* CA-CC. *Glutathione S-transferase epsilon 7* (*GSTe7*, FDR *p* = 1.64E-03), *cytochrome c oxidase subunit 7A1* (*COX7A1*, FDR *p* = 9.78E-04), and *arrestin domain-containing protein 3* (*ARRDC3*, FDR *p* = 2.26E-03) were the most down-regulated, with FCs of -1040, -1001 and − 713 (Table S5). These three genes were expressed in *WT Ae. aegypti* CA-CC but were barely detectable in *epox*^*−/−*^ glands. The expression pattern of the three biological replicates was also uniform. Among the top 10 genes with the largest max group mean, 8 were up-regulated and 2 were down-regulated.

The results of data analysis indicate that the knock-down of *epox* significantly altered the expression of several genes in the CA-CC, with a Log_2_ fold change of greater than 2, and false discovery rate below 0.05. Some of the most significantly differentially expressed genes include *Broad complex* (*Br-C*) *core protein*, *ADP/ATP carrier protein 2* (*AAC2*), *Troponin T* (*TpnT*), *muscle LIM protein 1* (*MLP1*), and *Lethal (2) essential for life* (*l(2)efl*) (Table S5). Several of these differentially expressed genes have been associated with the JH synthesis or signaling pathways. *Br-C* is significantly elevated by 4.31 folds in *epox*^*−/−*^ mutants. *Br-C* is a transcription factor that is responsive to JH. Its main function is to facilitate ecdysis and metamorphosis through the induction of ecdysone synthesis [48]. It is repressed by *Kr-h1*, and as a result, it is repressed by JH. It is also an ecdysone-inducible gene that suppresses the synthesis of JH. Therefore, it is an essential component within the JH feedback network [49]. The *epox*^*−/−*^ mosquitoes have high *Br-C* expression, but surprisingly, *Kr-h1*, which is the main inhibitory gene of *Br-C* and a major inductively responsive gene to JH, remains almost unchanged, with log_2_ fold change = -0.04 and FDR *p* = 0.92. One possible factor that contributes to the overexpression of *Br-C* is the *ecdysone receptor/ultraspiracle* (*EcR/USP*) dimer; in which *USP* is up-regulated by 0.323-fold with FDR *p* = 0.14, while *EcR* remained almost unchanged, with fold change = -0.135 and *FDR p* = 0.69.

*Takeout* (*TO*) was down regulated by -4.69 fold with FDR *p* = 1.90E-07 (Table S5). The *TO* gene encodes a JH binding protein that is highly expressed in *Diploptera punctata* and *Ae. aegypti* CAs [50], and it has been proposed that acts as an intracellular JH or JH precursor carrier protein [51]. The JH receptor, *Methoprene-tolerant* (*Met*), which is downstream of the JH regulatory pathway induces *TO* expression [52, 53].

Another differentially expressed gene that is involved in JH signaling pathway is *Nuclear Receptor Seven Up* (*SVP*), which was up-regulated by 0.485 folds with FDR *p* = 0.012 (Table S5). SVP plays a central role replacing *Ae. aegypti* USP in the AaEcR ⁄AaUSP heterodimer complex, thereby blocking the action of 20E [54]. The overexpression of SVP may be part of a JH synthesis feedback network, where the CA-CC of *epox*^−/−^ tries to produce more MF in response to a low JH signal. A similar JH feedback on JH synthesis has been recently described in *D. melanogaster* [55]. *Tailless* (*tll*) was up-regulated by 6.42-fold, although with suboptimal *FDR P* = 0.11 (Table S5). *tll* encodes a nuclear hormone receptor and is known to facilitate development in *D. melanogaster*, including larval segmentation [56], neurogenesis [57], and more importantly, the development of *corpora cardiaca* [58]. Its overexpression may increase CA-CC activity in response to a decreased JH signaling.

Among the up-regulated genes, *Nrf-6* is a membrane lipid transporter protein that has been extensively studied in *Caenorhabditis elegans.* It plays a crucial role in facilitating the cellular uptake of various nutrient molecules [59]. The *D. melanogaster beltless* (*blt*) gene, which is homologous to *Nrf-6*, supports oogenesis and embryogenesis via transportation of yolk proteins [60]. It is abundant not only in reproductive glands, but also in neuronal systems such as brain, ventral cord, neuro secretory cells and interneurons [61]. *Nrf-6* was found to mobilize small lipophilic molecules to surrounding tissue and most importantly, *Nrf-6* is responsible for lipid signaling in *C. elegans* [62]. This upregulation of *Nrf-6* in CA-CC may indicate an increase of CA-CC energy consumption required for more MF production, or it could imply altered downstream lipid signaling.

Epsilon-class *GST* (*GSTe*) genes are insect-specific, and they are known to confer pesticide resistance to *Ae. aegypti* and *Anopheles funestus* mosquitoes [63, 64]. However, a more important function of *GSTe7* is to facilitate insect molting and development under the regulation of ecdysone. A study in *B. mori* suggests that *GSTe7* expression was increased in correlation with high ecdysone titer [65], and its loss-of-function resulted in lethality during molting. The low JH signal in *Ae. aegypti* might increase the titer of ecdysone, therefore *GSTe7* is predicted to be overexpressed in *epox*^*−/−*^ mutants. However, we observed the exact opposite, where *GSTe7* was down-regulated by over a thousand-folds, being the most down-regulated gene in the library.

*AAC2* was up-regulated by 8-folds in CA-CC of *epox*^−/−^ (Table S5). It is an anti-apoptotic gene and plays cyto-protective roles in some cancer cells. It contributes to the maintenance of mitochondrial membrane integrity, preventing the onset of the intrinsic apoptosis pathway [66]. Its overexpression may result in a more active CA-CC. Moreover, *AAC*s are essential in providing metabolic energy during the flight of insects, and they are highly expressed in myofibrils of indirect flight muscles in *D. melanogaster* [67]. Overexpression of these proteins may indicate accelerated metabolic rate of CA-CC in *epox*^*−/−*^ insects [68, 69].

*COX7A1* is a subunit of cytochrome C oxidase, which is a tumor suppressor gene expressed in mitochondria [70]. It initiates the intrinsic apoptosis pathway, in which cytochrome C is released from mitochondria and activates caspase cascades. On the other hand, *ARRDC3* is a tumor suppressor gene in mammalian models, but it is conserved in insects such as *D. melanogaster* [71, 72]. It is a pro-apoptotic receptor expressed on cell surfaces, and interacts with receptors such as neural precursor development downregulated protein 4 (NEDD4) and β2-adrenergic receptor (β2AR) [73]. However, the more important role of ARRDC3 in regulating cell fate is to inhibit Yorkie (Yki), an essential protein in the Hippo pathway that promotes cell proliferation. The down regulation of pro-apoptotic genes such as *COX7A1* and *ARRDC3*, together with the up-regulation of proto-oncogenes such as *AAC2* might be related to dysregulated proliferation of *epox*^*−/−*^ CA cells.

### Interaction of differentially expressed genes and miRNAs

**Fig. 6:**
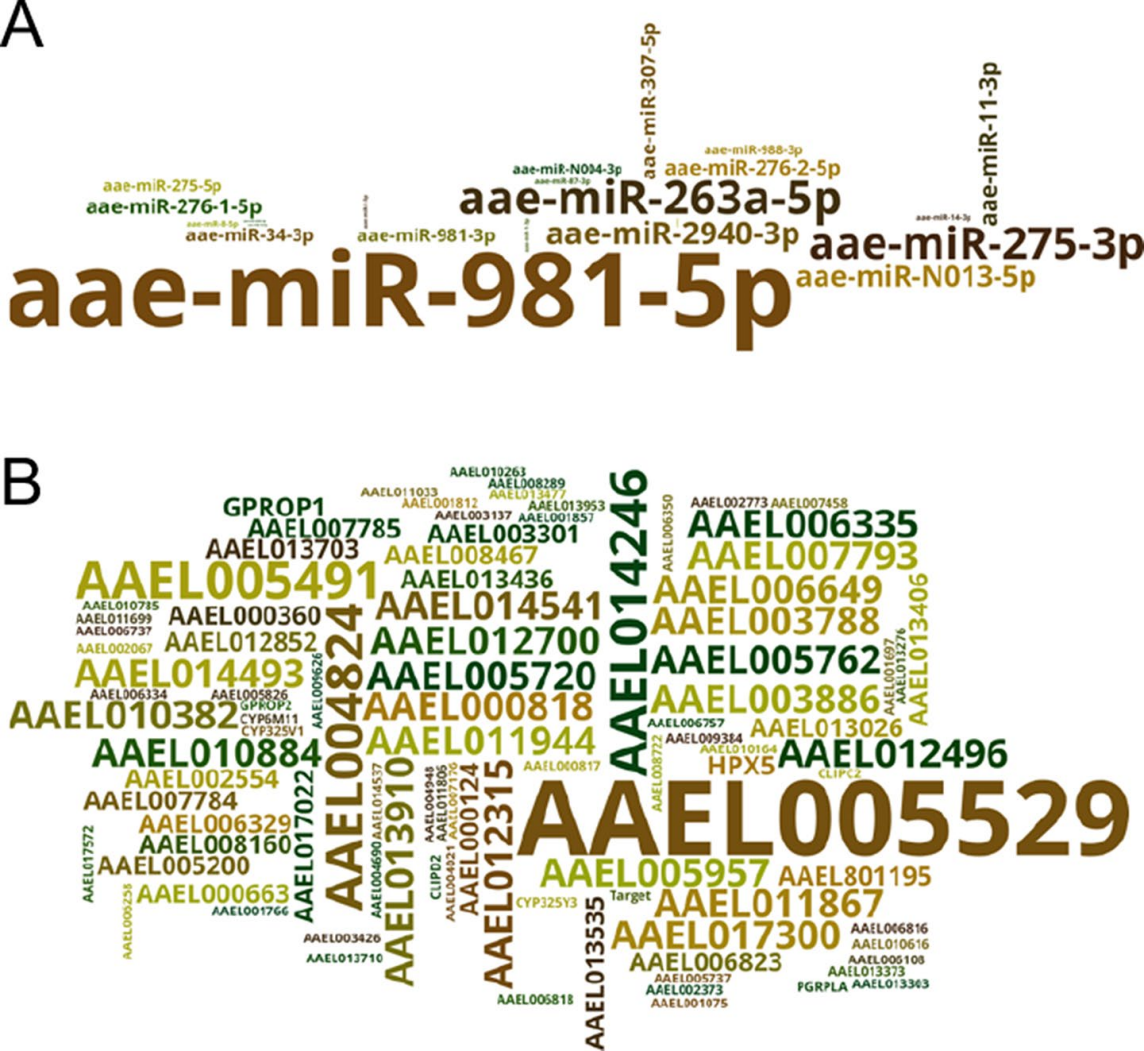
Visualization of interaction of differentially expressed genes and miRNAs. Genes (**A**) and miRNAs (**B**) visualized by number of binding sites

We used three different tools, namely RNA22, miRanda, and RNAhybrid, to predict the potential miRNAs’ interaction target sites on the differentially expressed genes. Sixteen differentially expressed genes were predicted to have at least one potential binding target by more than one software (Table 1). The number of binding sites for each miRNA and gene are visualized in Fig. 6A and B. It is predicted that aae-miR-981-5p, aae-miR-263a-5p, and aae-miR-275-3p regulate the highest number of genes, while *Futsch* is regulated by the highest number of miRNAs. A further investigation of these miRNAs and *Futsch* will most likely reveal their potential role in CA-CC homeostasis.

**Table 1:** Genes with miRNA binding sites predicted by more than one miRNA target identification tool

Gene Accession code	Description	miRNA binding site	miRNA(s)
AAEL005529	microtubule-associated protein futsch, transcript variant X8	8	aae-miR-11-3p, aae-miR-2940-3p, aae-miR-8-5p,
AAEL014246	UDP-glucuronosyltransferase 2B1, transcript variant X2	4	aae-miR-1-5p
AAEL007793	Alkyldihydroxyacetonephosphate synthase	3	aae-miR-275-3p
AAEL012496	ankyrin repeat and BTB	3	aae-miR-981-5p
AAEL003886	arrestin domain-containing protein 17	3	aae-miR-275-3p
AAEL017300	mucin-5AC, transcript variant X1	3	aae-miR-981-5p
AAEL005720	NACHT and WD repeat domain-containing protein 2	3	aae-miR-981-5p
AAEL003788	protein msta, transcript variant X1	3	aae-miR-981-5p
AAEL014541	protein-glucosylgalactosylhydroxylysine glucosidase	3	aae-miR-981-3p
AAEL006649	TNF receptor-associated factor 4	3	aae-miR-263a-5p
AAEL002554	anosmin-1, transcript variant X1	2	aae-miR-981-5p
AAEL017022	cell wall protein DAN4, transcript variant X1	2	aae-miR-275-5p
AAEL000360	dnaJ homolog subfamily B member 13	2	aae-miR-263a-5p
AAEL000663	MAPK regulated corepressor interacting protein 2	2	aae-miR-11-3p
AAEL012852	trypsin 3A1-like	2	aae-miR-11-3p
AAEL005200	venom carboxylesterase-6, transcript variant X4	2	aae-miR-981-5p

A two-model target analysis showed that miR-981-5p has 40 potential target predictions on 31 different genes, ranked at the top by number, while miR-263-5p and miR-275-3p have 18 and 17 potential target sites on 15 and 13 genes, respectively (Table S8). Other miRNAs with potential target sites include miR-34-3p, miR-2940-3p, miR-11-3p, miR-N013-5p, miR-276-1-5p, miR-981-3p, miR-N004-3p, miR-988-3p, miR-307-5p, miR-275-5p, miR-14-3p, miR-87-3p, miR-1-3p, miR-210-5p, miR-184-5p, miR-210-3p, miR-8-5p, miR-2942-3p, miR-10-5p, miR-1-5p, with 1–6 potential targets (Table S8).

Among all the genes that have been identified with miRNA binding sites, the gene with the highest miRNA match number is *microtubule-associated protein* (*MAP*) *Futsch.* Its interactive miRNAs include miR-11-3p, miR-263a-5p, miR-2940-3p, miR-8-5p, and miR-981-5p, with miR-11-3p predicted by both miRanda and RNA22, miR-2940-3p and miR-8-5p predicted by RNA22 and RNAHybrid (Table S9). The gene was up-regulated by 5.47-fold in CA-CC of *epox*^*−/−*^. *Futsch* is a microtubule binding protein that maintains microtubule loops as well as the tubulin mass. It is known to regulate synaptic growth in *D. melanogaster* [74]. As described above, miR-11-3p and miR-263a-5p facilitate insect neuronal development. While the binding target between *Futsch* and miR-263a-5p was predicted by miRanda only, both RNAhybrid and miRanda predicted binding between *Futsch* and miR-11-3p, which were both up-regulated in *epox*^*−/−*^. The binding between *Futsch* and miR-8-5p/miR-2940-3p was predicted by RNAhybrid and RNA22. Consequently, the association between miR-11-3p, miR-8-5p, miR-2940-3p and *Futsch* is worth investigating.

The main function of *UDP-glucuronosyltransferase* (*UGT*) is to metabolize xenobiotic compounds into non-toxic substances, more specifically through the catalysis of lipophilic compound glycosylation [75–77]. It is closely associated with insecticide resistance [78–80], and confers resistance to temperature stress. It is also believed that some genes in the *UGT* family regulate transmembrane transportation, while others are receptors responding to exterior stress signals [81]. The gene was down-regulated in *epox*^*−/−*^ by 5.1-fold and is potentially targeted by miR-1-5p, miR-2940-3p, and miR-988-3p. Both RNA22 and RNAhybrid found potential target sites for miR-1-5p, while miR-2940-3p and miR-988-3p were predicted by RNAhybrid only (Table S9).

*ATP-binding cassette transporters* (*ABC)* are a class of membrane-bound ATP-dependent pumps [82]. *Class G ABC* (*ABCG*) is highly expressed in prothoracic gland, where ecdysone is synthesized. It functions in the transportation of ecdysone and thus participating in the synthesis of 20E [83]. In addition, it also functions in xenobiotic detoxification [84]. It is down-regulated in *epox*^*−/−*^ mutants by 244.7-fold. miR-276-1-5p, miR-276-2-5p, miR-2940-3p and miR-981-3p were predicted to target *ABCG20*. miR-276-1 and miR-276-2 are generated from distinct precursor loci but have the same mature sequence, and all four matches were predicted by RNAhybrid (Table S9).

*Alkyldihydroxyacetonephosphate synthase* (*AGPS*) regulates neuronal development in *D. melanogaster* [85]. It is targeted by miR-275-3p and miR-981-5p. Both miRanda and RNAhybrid predicted target binding between miR-275-3p and *AGPS*, while miR-981 was predicted only by RNA22 (Table S9). The role *APGS* may play in CA-CC metabolism remains to be elucidated.

## Conclusions

This work presents a comprehensive analysis of the transcriptome and small RNA profiles of wild-type and CRISPR-Cas9 mediated mutation of the *epoxidase* gene in *Ae. aegypti*. Epoxidase is a key enzyme in the synthesis of juvenile hormone. Experimental work with *corpora allata* is very challenging due to their very small size and therefore producing transcriptomes of these specialised glands is very valuable in itself. While the presented work is mainly descriptive, it provides comprehensive transcriptome and small RNA profiles of the glands in an important insect vector, as well as insights into the impact of JH signalling on the CA-CC biology.

### Electronic supplementary material

Below is the link to the electronic supplementary material.

**Supplementary Material 1:** **Fig. S1.** Venn diagram of up-regulated (Left) and down-regulated miRNAs (Right) in CA inactive vs CA active stage or strain of *Ae. aegypti*

**Supplementary Material 2:** Supplementary Tables 1–9

## Data Availability

The datasets for the small RNA and transcriptome libraries used in this article are available in the NCBI repository with the SRA and the BioProject ID PRJNA1034688.
