# Supplementary material for: Differential gene expression and microRNA profile in corpora allata-corpora cardiaca of Aedes aegypti mosquitoes with weak juvenile hormone signalling
Source: BMC Genomics. 2024 Jan 25;25:113. doi: 10.1186/s12864-024-10007-9 (PMC10811912; doi:10.1186/s12864-024-10007-9)
Supplement: Supplementary file 1 — Supplementary Material 1: Fig. S1. Venn diagram of up-regulated (Left) and down-regulated miRNAs (Right) in CA inactive vs CA active stage or strain of Ae. aegypti [file 12864_2024_10007_MOESM1_ESM.pdf]

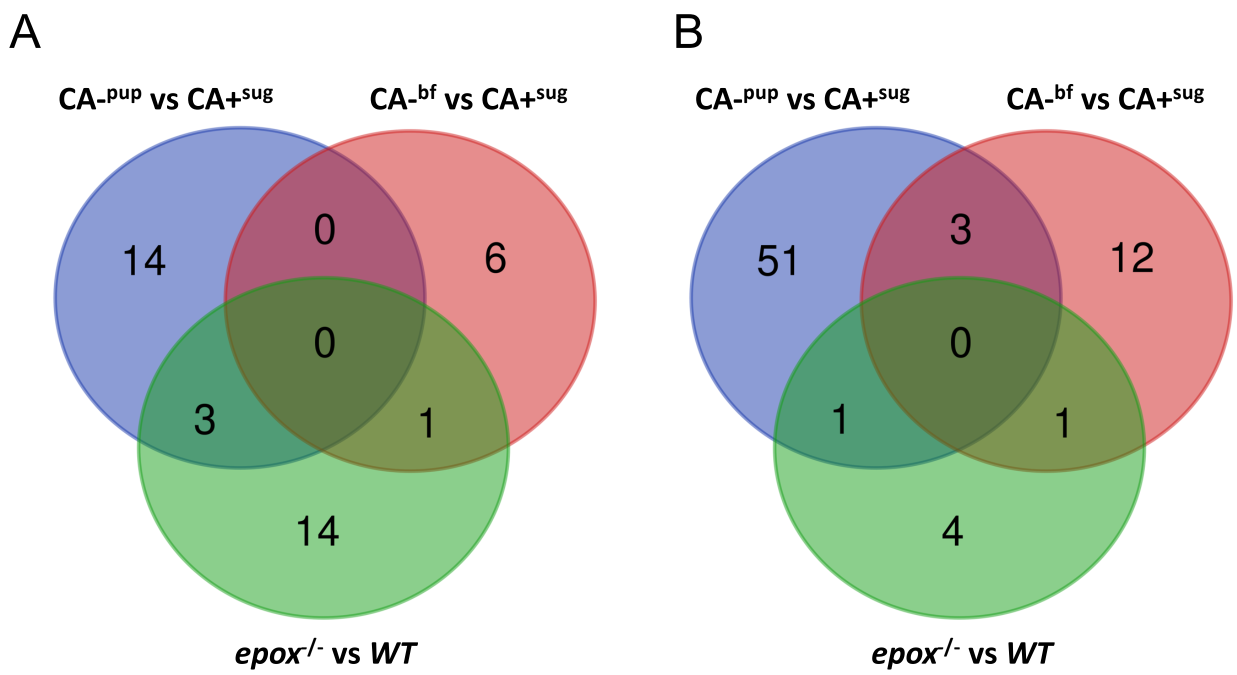

Fig. S1: Venn diagram of up-regulated (Left) and down-regulated miRNAs (Right) in CA inactive vs CA active stage or strain of *Ae. aegypti*.
